# The MacBrain Resource Center (MBRC) rhesus macaque postnatal brain histology datasets: Enabling new discoveries through NHP tissue and digital data Repositories

**DOI:** 10.1111/joa.70183

**Published:** 2026-06-04

**Authors:** Valeria Mendoza‐Silva, Lucy Greene, Emma Burke, Cristina Canales, Julia Chocarro, Jose L. Lanciego, Khuleshwari Kurrey, Phil Barello, Aya Nusir, Nadine Kabbani, Kathleen S. Rockland, Dibyadeep Datta, Jon I. Arellano, Amy F. T. Arnsten, Yury M. Morozov, Bashir Ahmed, Zoltán Molnár, Caroline J. Zeiss, Pasko Rakic, Alvaro Duque

**Affiliations:** ^1^ Department of Neuroscience, MacBrain Resource Center (MBRC) Yale University School of Medicine New Haven Connecticut USA; ^2^ CNS Gene Therapy Program, Center for Applied Medical Research (CIMA) University of Navarra Pamplona Spain; ^3^ Centro de Investigación Biomédica en Red de Enfermedades Neurodegenerativas (Ciberned‐ISCIII) Madrid Spain; ^4^ Aligning Science across Parkinson's (ASAP) Collaborative Research Network Chevy Chase Maryland USA; ^5^ Interdisciplinary Program in Neuroscience George Mason University Fairfax Virginia USA; ^6^ School of Systems Biology George Mason University Fairfax Virginia USA; ^7^ Department of Anatomy & Neurobiology Boston University Chobanian and Avedisian School of Medicine Boston Massachusetts USA; ^8^ Department of Neuroscience Yale University School of Medicine New Haven Connecticut USA; ^9^ Department of Psychiatry Yale University School of Medicine New Haven Connecticut USA; ^10^ Department of Physiology, Anatomy and Genetics University of Oxford Oxford UK; ^11^ Department of Comparative Medicine Yale University School of Medicine New Haven Connecticut USA

**Keywords:** autoradiography, databases, electron, factual, immunohistochemistry, *macaca mulatta*, microscopy, tissue banks

## Abstract

In our companion paper, in this issue, we describe our efforts to provide embryonic rhesus macaque (*Macaca mulatta*) brain histology for the study of brain development, with emphasis on cortical development. Here we continue our description of efforts to provide postnatal rhesus macaque brain histology relevant for the study of cellular circuits which continue to mature and change well into postnatal life, in males and females, and which naturally deteriorate in the elderly. The mission of the MacBrain Resource Center (MBRC) in the Department of Neuroscience at Yale University School of Medicine is to provide a cost‐effective means for researchers to conduct de novo studies on this non‐human primate (NHP) brain animal model using materials already in existence and therefore without exorbitant costs and without having to sacrifice additional animals (https://medicine.yale.edu/neuroscience/macbrain/mission/). Here we report on how this mission is being accomplished. Because MBRC materials have been and continue to be gathered from unrelated studies over many years, most methods have already been published. The MBRC divides different types of materials into separate Collections. The present description of histo‐ and immunohistochemical processes is limited to current work that provides materials to populate Collection 6 and is accurate as of May 2026. Collections in the MBRC are dynamic. Of the 8 current MBRC datasets, here we emphasize Collections 5, 6, and 7 and illustrate through examples how different materials are currently being used to conduct research both in cortical and subcortical structures. Many of the electron microscopy (EM) blocks in Collection 5 sampling the brain at numerous regions come from the >100 cases of titrated thymidine (^3^H‐TdR) injections in Collection 1 in addition to cases in Collections 2 and 3. Altogether, at present there are ~1000 inventoried EM blocks collected from postnatal cases. Collection 6 currently contains >30,000 digital images illustrating 35 different cellular and fiber markers in 32 brains of both sexes ranging from P0 to 32 years of age. Materials in Collections 6 and 7 keep growing as we constantly process and add NHP brains to them. Based on the molecular, genetic, and anatomical similarities between this animal model and human, we underline the importance of archiving and (re‐)using rhesus macaque brains to foster neuroscience research. As far as we know the NHP brain materials in the MBRC Collections constitute the largest datasets of their kind in the world.

## Graphical abstract

The MacBrain Resource Center (MBRC) postnatal rhesus macaque brain histology datasets are organized into Collections that promote cost‐effective de novo research. This article provides examples from Collections 5, 6, and 7 and describes the histo‐ and immunohistochemical (IHC) processing of materials for Collection 6. Collection 5 includes postnatal electron microscopy blocks mostly from ^3^H‐TdR cases in Collection 1. Collection 6 offers zoomable and downloadable images of >30,000 stained coronal sections from, so far, 32 M & F brains aged P0 to 32 years. Collection 7 contains tissue blocks available for further processing. Public digital access of brain‐wide histological references fosters use and re‐use of archived tissue increasing scientific rigor and transparency while reducing animal sacrifice.

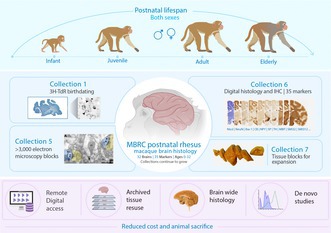

## 1 INTRODUCTION

To understand the mechanisms responsible for brain changes across the lifespan, and whether regions or regional changes differ in males (M) and females (F), is essential to identifying and understanding the pathological vulnerabilities associated with different critical periods, sex, ageing, and to study evolutionary trends. While non‐primate species, cell lines, organoids, genetically modified animals, and other in vitro and in vivo models provide valuable data, they cannot fully replace the need for understanding normal histological differences between sexes, or in the same animal at different ages, in a species with high homology to human and hence with the highest translational value (Birtele et al., 2025; Urrestizala‐Arenaza et al., 2024).

This opportunity is afforded by non‐human primates (NHP) that are evolutionarily, anatomically, and molecularly very close to us. For these very reasons, of the multiple NHPs available for research, the rhesus macaque (*Macaca mulatta*) is the most used species in biomedical research (Gibbs et al., 2007; Hannibal et al., 2017; Mattison & Vaughan, 2017). The homology between rhesus and human is high at every level from the genetic, cellular and systems attributes to the basic animal behavioral phenotypes (Acharya & Byrareddy, 2024; Chiou et al., 2023; Gibbs et al., 2007; Lu et al., 2024; Mahyari et al., 2025; Warren et al., 2020) and these, in addition to a gyrencephalic brain, long gestational period, and ~1/3 the lifespan of the human, offer unique experimental opportunities not available in other species and that for obvious reasons, are not, and cannot be made, available in humans (Dash et al., 2023; Duque, 2023; Duque et al., 2025; Nakamura et al., 2021; Rushmore et al., 2021).

However, one practical problem is that collecting primate tissue is expensive and slow (Mattison & Vaughan, 2017). These challenges make it difficult for single laboratories to create large and complete datasets that include both Ms and Fs of different ages using uniform methods covering distinct histo‐ and immunohistological markers. The logistic constraints and the high value of the data justify the need for shared reference resources. This is one of the main drivers for the creation of our datasets. Being able to explore the brain cellular information our data sets contain, through the lifespan of this very close NHP relative of ours, is fundamental to the effective use of this animal model to recapitulate how key biological aspects change under normal aging. We consider understanding these normal differences fundamental and primordial to understanding those that are pathological.

Rhesus macaques are used in studies of, for instance, Alzheimer's disease (AD) (Datta et al., 2021; Leslie et al., 2021; Zeiss, Duque, & Huttner, 2025; Zeiss, Huttner, et al., 2025), Parkinson's disease (PD) (Chocarro et al., 2023; Rico et al., 2024), Tourette's syndrome (McCairn et al., 2009; Worbe et al., 2013), and schizophrenia (Goldman‐Rakic, 1994, 1999; Goldman‐Rakic & Selemon, 1997). Our extensive collections of histological data will help interpret results and foster and guide further experiments in these diseases and a host of additional maladies that affect humans. For instance, the data will help explore why the incidence of AD increases significantly with age and is about twice as common in F than in M, while the effect of sex is the opposite in PD, another neurodegenerative disease in which increasing age is a principal factor, but which affects about twice more M than F (Aggarwal & Mielke, 2023; Beam et al., 2018; Hirsch et al., 2016). In comparison, illnesses like Tourette's syndrome appear not in the elderly but in children of school age, affect three to four times more M than F, and instead of getting worse with age, in many cases symptoms show improvement in adolescence (Baizabal‐Carvallo & Jankovic, 2023; Robertson et al., 2017). Following a different tempo, the first episode of schizophrenia usually occurs in late teenage years, and its prevalence is about equal between M and F, although of course, there are significant differences in incidence rates, age at onset, severity of positive, and negative symptoms, etc. (Kleinhaus et al., 2011; Salehi et al., 2024). Our histological data can provide a better grasp on the cellular circuits and mechanisms compromised in these complex human brain disorders and the unique behaviors that accompany them by affording more precise knowledge of the neuronal and glial components that orchestrate the normal physiology of neuronal networks in this NHP model.

Currently, the MBRC houses 8 different Collections. In short, Collection 1 contains embryonic and postnatal brain tissue from tritiated thymidine (^3^H‐TdR) injections in pregnant females from very early (~30 days) to very late (~160 days) gestational ages and with sacrifice of the fetus or offspring at different survival periods after injection, varying from about 1 h to several years. Collection 2 has materials from tract tracing injections. Collection 3 consists of prenatal and postnatal lesions. Collection 4 has materials gathered after prenatal X‐ray irradiation. Collection 5 has EM blocks prepared from many different regions and ages, on materials originally prepared mostly for Collections 1 and 2. Collection 6 has histo‐ and immunohistochemical materials from, as of the time of writing this report, 35 different cellular and fiber markers (Table 1) used in 32 brains. Collection 7 consists of brain tissue blocks; preserved frozen and already tested for histochemical viability. Most of the materials in this collection were fixed with 4% paraformaldehyde (PFA), a few also contained glutaraldehyde (GA), and a few more have been collected as fresh unfixed tissue. The number of samples collected in this last category (fresh tissue) is increasing because experiments with this requirement that contribute tissue to the MBRC are ongoing. Collection 8 is the latest addition to the datasets and consists of materials gathered in conjunction with the Alzheimer's Disease Research Center (ADRC) at Yale and hence contains elderly NHP materials specifically used in AD research as well as relevant examples of human materials. Further details on the contents of each Collection can be found in the MBRC website at https://medicine.yale.edu/neuroscience/macbrain/.

**TABLE 1:** Histo‐ and immunohistology abbreviations.

Abbreviation	Definition
AChE	Acetylcholinesterase
Iron	Iron
Nissl	Nissl substance
5‐HT	Serotonin
a‐Syn (SNCA)	Alpha‐synuclein (SNCA 4D6)
b‐Amy(6E10)	Beta‐Amyloid, clone 6E10
CB	Calbindin
CCK	Cholecystokinin
ChAT	Choline acetyltransferase
CR	Calretinin
GABA	Gamma‐aminobutyric acid
DBH	Dopamine beta‐hydroxylase
GABA‐B	Gamma‐aminobutyric acid type B receptor
GFAP	Glial fibrillary acidic protein
Iba1	Ionized Ca2+ binding adaptor molecule 1
LTX	Latexin
MBP	Myelin basic protein
NeuN	Neuronal nuclear protein OR Fox3
NOS	Nitric oxide synthase
NPY	Neuropeptide Tyrosine
NRGN	Neurogranin
Nurr1	Nuclear receptor related 1 protein
Olig2	Oligodendrocyte transcription factor 2
PV	Parvalbumin
S100	S100 calcium binding protein family
SATB2	Special AT‐rich sequence binding protein 2
SMI‐312	Phosphorylated neurofilament protein pan axonal cocktail
SMI‐32	Non‐phosphorylated neurofilament protein 32
SOM	Somatostatin
SP	Substance P
SV2a	Synaptic vesicle glycoprotein 2A
TauAT8	Hyperphosphorylated microtubule‐associated protein tau, Ser202, Thr205
TauP217	Hyperphosphorylated microtubule‐associated protein tau, Thr217
TBR1	Brain Specific T‐box transcription factor 1
TH	Tyrosine hydroxylase

The principal objectives of this study are (1) To report on the postnatal materials currently available in the MBRC. (2) To illustrate the methodologies used to acquire, store, process, digitize and make public those materials and provide details of the histo‐ and immohistology used in preparation of Collection 6. (3) To provide examples of how materials in Collections 5, 6, and 7 are currently being used for de novo research. (4) To discuss other public databases containing similar NHP brain materials and to broadly compare their contents to those in the MBRC Collections. As in our companion paper on embryonic datasets, here our long‐term goals for postnatal datasets are also severalfold. We strive for our postnatal materials to include sufficient data to establish a normative standard for NHP studies and we are making an effort to process and make public high‐resolution cytoarchitectonic data against which imaging obtained by any means can be compared or registered. The availability of our postnatal datasets will ensure data sharing and reusability that will foster NHP brain studies, increase research transparency and decrease not only the cost of research but very importantly the number of animals needed to be sacrificed in future studies.

## 2 MATERIALS AND METHODS

Detailed methods have been published over the last 50 years for all the materials archived in all Collections. The methodologies encompass from injections and surgical procedures (e.g., tracer injections, lesions, C‐sections, enucleations) to diverse staining and visualization methodologies including autoradiography, Golgi staining, transmitted and fluorescence microscopy, and EM (Ahmed et al., 2024; Duque et al., 2016, 2025; Kostovic & Rakic, 1980; McDonald & Duque, 2022; Morozov & Rakic, 2024; Rakic, 1971, 1972, 1974; Rakic 1988; Rakic et al., 1988; Sakharkar et al., 2022; Selemon et al., 2013; Spadory et al., 2022). For methods employed to obtain or process materials in the newer Collection 8 see (Zeiss, Duque, & Huttner, 2025; Zeiss, Huttner, et al., 2025). Further details regarding timed pregnancy logistics in the rhesus macaque breeding colony, brain cutting and storage (e.g., Collection 6), general histology, etc., can be found in (Duque et al., 2025). Any questions can be directed to the corresponding author. The following limited description covers general and common protocols for histo‐ and immunohistochemistry used in recent years to populate Collection 6 in addition to antibody related details.

### 2.1 Animals

Many of the animals used in the production of Collection 1 (^3^H‐TdR) and hence Collection 5 (EM blocks) date from the late 1960s and early 1970s while Dr. Pasko Rakic was still at Harvard. Most subsequently obtained and/or processed materials in those and other Collections (1980s to present) have been gathered at Yale University. Independent of location, all animal studies were conducted in accordance with federal and state regulations and were reviewed and approved by the corresponding Institutional Animal Care and Use Committee (IACUC). In the last 20 years, most cases that contributed, and still contribute, complete hemispheres or tissue blocks to Collections 6 and 7 consist of rhesus macaques transcardially perfused with phosphate buffered saline (PBS) followed by 4% PFA in PBS with most brains postfixed overnight in the same fixative. For EM analysis (Collection 5) animals were transcardially perfused with a mixture of 4% PFA and 0.5%–1% glutaraldehyde in PBS, and brains were postfixed with 1% osmium tetroxide, dehydrated, and embedded in Epon‐Araldite epoxy resin. Brain hemispheres are commonly separated at the midsagittal plane and immersed in 20% and then 30% sucrose (in PBS) for cryoprotection. All materials in the MBRC brain bank are left from unrelated studies. In addition, fresh, unfixed tissue collected for molecular analysis currently contributes materials to the MBRC.

### 2.2 Tissue storage and cutting

After sucrose cryoprotection, many whole hemispheres or tissue blocks were stored at either −20 or −80°C. To populate Collection 6, samples in which at least one hemisphere was complete were either processed in‐house or sent to FD Neurotechnologies (Columbia, MD) for processing. Independent of location, all samples were processed nearly identically. The samples sent to FD were sent via overnight priority shipping at room temperature (RT), wrapped in gauze (non‐woven 4″ × 4″ sponges), and sealed in a 250‐mL Nalgene jar filled with cold 20%–30% sucrose in PBS. So far, most brain hemispheres have been cut in the coronal plane at 50 μm on a freezing microtome and sections stored in cryoprotectant solution (25% glycerol and 30% ethylene glycol by volume in PBS, or FD tissue cryoprotection solution™) until further processing. Sequential sections were histologically stained with Nissl or reacted for acetylcholinesterase (AChE) or iron or, alternatively, immunohistochemically processed. During optimization, different antibodies for the same marker but manufactured by different companies, or with different primary hosts, and/or at different dilutions may have been tried. The antibodies and dilutions that we empirically determined to be best, and therefore the ones we continue to use, are listed in Table 2. Further details can be requested from the corresponding author.

**TABLE 2:** List of antibodies.

Primary antibodies	Host	Description	Catalog #	RRID	Source/company	Dilution
5‐HT	Rabbit	Polyclonal	20080	AB_572263	ImmunoStar (IBL)	1:10K
a‐Syn (SNCA)	Mouse, clone 4D6	Monoclonal	834304	AB_2734600	BioLegend	1:5000
b‐Amy (6E10)	Mouse, clone 6E10	Monoclonal	803014	AB_2728527	BioLegend	1:2500
CB	Mouse	Monoclonal	C9848	AB_476894	Millipore‐Sigma	1:1000
CCK	Rabbit	Polyclonal	MBS555736	NA	MyBioSource	1:4000
ChAT	Mouse, clone CL3173	Monoclonal	NBP2‐46620	AB_2922998	NOVUS	1:500
CR	Rabbit	Polyclonal	AB5054	AB_2068506^a^	Millipore	1:20 K
CR	Rabbit	Polyclonal	CR7697	AB_2619710	Swant	1:10 K
DBH	Rabbit 2B11	Monoclonal	ZRB1328	NA	Millipore‐Sigma	1:1000
GABA	Rabbit	Polyclonal	20,094	AB_572234	ImmunoStar	1:10 K
GABA‐B	Mouse	Monoclonal	ab55051	AB_941703	Abcam	1:20 K
GFAP	Rabbit	Polyclonal	Z0334	AB_10013382	Agilent‐DAKO	1:5000
Iba1	Rabbit	Polyclonal	019–19,741	AB_839504	FujiFilm Wako	1:8000
LTX	Mouse	Monoclonal	MA5‐25742	AB_2724584	Thermo Fisher	1:250
MBP	Rat, clone 12	Monoclonal	MAB386	AB_94975	Millipore‐Sigma	1:200
NeuN	Mouse	Monoclonal	MAB377	AB_2298772	Millipore‐Sigma	1:3000
NOS	Rabbit	Polyclonal	61–7000	AB_2313734	Thermo Fisher	1:1000
NPY	Rabbit	Polyclonal	N9528	AB_260814	Sigma‐Aldrich	1:8000
NRGN	Rabbit	Monoclonal	ab230154	NA	Abcam	1:4000
Nurr1	Mouse	Monoclonal	ab41917	AB_776887	Abcam	1:250
Olig2	Rabbit	Polyclonal	AB9610	AB_570666	Millipore‐Sigma	1:4000
PV	Mouse	Monoclonal	P3088	AB_477329	Millipore‐Sigma	1:2500
S100	Rabbit	Monoclonal	ab52642	AB_882426	Abcam	1:2000
SATB2	Mouse	Monoclonal	ab51502	AB_882455^b^	Abcam	1:250
SMI‐312	Mouse	Monoclonal	837904	AB_2566782	BioLegend	1:1000
SMI‐32	Mouse	Monoclonal	801701	AB_2564642	BioLegend	1:1000
SOM	Sheep	Polyclonal	20C‐CR2056SP	AB_1288773	Fitzgerald	1:3000
SP	Rat	Monoclonal	sc‐21,715	AB_628299	Santa Cruz	1:250
SV2a	Mouse	Monoclonal	sc‐376234	AB_10988568	Santa Cruz	1:5000
TauAT8	Mouse	Monoclonal	MN1020	AB_223647	Invitrogen	1:5000
TauP217	Rabbit	Polyclonal	44–744	AB_2533741	Thermo Fisher	1:5000
TBR1	Rabbit	Polyclonal	AB31940‐1001	AB_2200219	Abcam	1:1000
TH	Rabbit	Polyclonal	P40101	AB_2617184	Pelfreeze	1:1000

^a^ Discontinued.

^b^ This antibody recognizes both SATB1 and SATB2, not SATB2 exclusively.

### 2.3 Histo‐ and immunohistochemistry

For Nissl histochemistry we mostly use cresyl violet. Sections are mounted on gelatin coated or Superfrost Plus (charged) glass slides and allowed to dry. Before staining they are defatted by passing them through a series of increasing graded dilutions of ethanol, about 3–5 min each. Then, they are rehydrated by passing them back into decreasing concentrations of ethanol until they reach double‐distilled water. Depending on how dark one may want to stain the sections, they are put into the Nissl solution for 5–30 min. The Nissl solution commonly consists of 0.1 M sodium acetate (SA), 0.2 M formic acid (FA), and 0.5% aqueous cresyl violet (ACV) mix in the following proportion: 30 mL + 150 mL + 300 mL of SA, FA, and ACV correspondingly. Following dehydration in ethanol, sections are cleared in xylene and coverslipped with Permount® (Fisher Scientific).

For acetylcholinesterase (AChE) free‐floating sections were processed using a copper thiocholine method following Hedreen et al. (1985) with primate implementation as in Green and Mesulam (1988). Briefly, after washes in 0.01 M PBS (pH 7.4), sections are pretreated in 0.05 M acetate buffer (pH 5.3) 3 × 3 min. Sections are then incubated in solution A, containing sodium acetate, copper sulfate, glycine, ethopropazine, and acetylthiocholine for 24 h. After rinsing, the sections are incubated in solution B containing sodium sulfide (pH 7.8), followed by incubation in a solution C containing silver nitrate. Afterwards, the sections are re‐fixed overnight in 0.1 M PBS (pH 7.4) containing 4% PFA and thoroughly washed in distilled water. Finally, the sections are mounted on gelatin coated or Superfrost Plus slides. Following dehydration in ethanol, sections are cleared in xylene and coverslipped with Permount® (Fisher Scientific). At FD, the sections are stained with FD cresyl violet solution™ and the results, as per our observation, are identical to what we obtained in‐house.

For iron, we used the Perl's Prussian Blue method for detection of ferric iron (Fe^3+^) following the manufacturer (FD Neurotechnologies) specifications (Kit catalog number SS602). The kit contains proper solutions of potassium ferrocyanide and hydrochloric acid which upon reacting with ferric iron in the tissue produce the typical blue ferric ferrocyanide (Prussian Blue).

For immunohistochemistry, all rinses and incubations are done on free‐floating sections in 0.1 M PBS at RT unless otherwise stated. Stored and cryo‐protected sections are thoroughly rinsed to remove the cryoprotectant solution and subsequently immersed in 0.2–0.5% hydrogen peroxidase (5–10 min) to inactivate endogenous peroxides. Parallel series of sections spaced 1 mm apart (i.e., one series repeating every 20 sections) are processed for the same antibody. For this, sections are incubated, with gentle agitation, for 48–72 h in a solution containing the primary antibody (Table 2) and 0.1%–0.2% Triton‐X. After rinsing, sections are incubated for 12–24 h in secondary biotinylated antibody, at 1:200–500 dilution with 2%–5% serum from the animal host of the secondary antibody (or bovine serum albumin), with a final incubation in avidin–biotin–peroxidase complex (Vectastain elite ABC kit, Vector Labs, Burlingame, CA, USA; 4 h). Label is visualized by 0.05% 3′,3′‐diaminobenzidine (DAB) as a chromogen, precipitated by 0.01% hydrogen peroxide. The DAB reaction protocol varies little from specimen to specimen or stain to stain and in more general terms is according to the avidin–biotin complex protocol provided by Vector laboratories (Burlingame, CA) and/or the method of Hsu and Raine (1981) with the Vectastain elite ABC Peroxidase kit. After three‐ to five thorough washes, all sections are treated as previously described, that is, mounted on glass microscope slides, dehydrated in ethanol, cleared in xylene, and coverslipped with Permount® (Fisher Scientific, Fair Lawn, NJ, United States). DAB immunoreacted sections are stored at RT. Results from many of the antibodies listed in Table 2 have already been described in our own studies (e.g., McDonald & Duque, 2022; Sakharkar et al., 2022; Zeiss, Duque, & Huttner, 2025; Zeiss, Huttner, et al., 2025) in addition to thousands of citations provided by the manufacturers. Data in the table have been summarized and are provided with the most current general information. If further or exact information is needed on the use of any particular antibody for any specific case (brain number), please contact the corresponding author. The caveats are that manufacturers or vendors discontinue products, change distributors, make changes that affect the catalog number of a product, etc. In addition, we may, for instance, change final dilution, add detergent, or increase or decrease final concentrations, adjust final development time, etc. Although homogeneity of processing is a norm, occasional adjustments are necessary to generate the best data possible.

### 2.4 Digitization, digital data storage, and public web galleries

Digitization of our postnatal materials is identical to that of our embryonic tissue. For further details, please see our companion paper in this issue (Duque et al., 2025; Mendoza‐Silva et al., 2026).

### 2.5 Use of the artificial intelligence platform Aiforia

Striatal ChAT^+^ cells were quantified using Aiforia. Following is a summary of the steps from image preparation, training, segmentation, to exported metrics. First, coronal sections mounted on glass slides were digitized by scanning them at 20× using an Aperio CS HR scanner (Leica). Digital sections were then available to be downloaded from the MBRC gallery as .svs files. Each image was individually cropped to contain the striatum (the region of interest, ROI) in Aperio ImageScope and saved as a .tif at the same magnification. Files were then processed in Photoshop to remove pixels outside the striatum (e.g., white matter, internal capsule, nucleus accumbens) at the same image scale (0.5045 μm/pixel), then uploaded to Aiforia. In the AI platform, the images were analyzed using a deep‐learning workflow that first segmented the image out from the background and then detected ChAT^+^ cells within the segmented tissue. A tissue/background model was trained on annotated examples and ran for 100 iterations. Following, it was asked to analyze images it had not seen before. This layer served as the background mask for the model of cell detection. For the cell detection, ChAT^+^ somata were annotated using a target stamp (radius 35 μm) with subsequent refinement using instance segmentation. Training and test analyses were iteratively performed, and training set annotations were adjusted to reduce labeling errors and exclude artifacts (e.g., blood vessels). The Aiforia platform automatically exported outputs that included an annotated .tif with striatal tissue area and a count of ChAT^+^ cells. These were then used to calculate the cell density as number of cells per unit area.

## 3 RESULTS

### 3.1 Available postnatal data in collection 6 of the MBRC

As of May 2026, histo‐ and immunohistochemistry has been done, and made publicly available, in *n* = 32 (13M & 19F) different brains which range in age from postnatal day 0 (P0, B91) to 32.42 years old (B95). From these *n* = 32 brains, 424 different series of sections (i.e., galleries) encompassing 35 different stains, have been prepared. So far, the minimum to the maximum number of specimens in which repeated stain series are available ranges from 3 (e.g., for Iron, a‐Syn, b‐Amy) to 32 (ChAT and NPY). The 424 galleries contain 30,498 already digitized and made public brain coronal sections. Additionally, series of sections that will soon become galleries are in the process of being digitized; these not yet public galleries contain 7371 coronal sections. The number of images per gallery ranges from 66 to 78. The smallest number (66 images) belongs to B91 which at P0 is the youngest and smallest brain. The largest number (78 images) belongs to B67 and B88, two adult F brains ages 10.08 and 10.22 years old. These data are summarized in Table 3 organized left to right by specimen age.

**TABLE 3:** Summary of NHP postnatal data available in Collection 6 of the MBRC as of May 2026.

Sample	1	2	3	4	5	6	7	8	9	10	11	12	13	14	15	16	17	18	19	20	21	22	23	24	25	26	27	28	29	30	31	32	Totals
Brain #	B91	B86	B64	B84	B69	B74	B61	B73	B66	B63	B65	B80	B81	B82	B89	B94	B96	B62	B83	B85	B87	B92	B93	B72	B67	B79	B88	B68	B124	B101	B176	B95	
Sex	F	M	F	M	M	F	M	M	F	F	M	M	M	M	F	F	F	M	F	M	M	F	F	F	F	F	F	F	F	M	F	F	13 M‐19F
Stain/age	P0	P1	P7	P8	P10	P74	P75	P75	P75	P77	P78	6 m	6 m	6 m	6 m	6 m	6 m	1 y	1 y	1 y	1 y	1 y	1 y	8 y	10 y	10 y	10 y	19 y	19 y	24 y	27 y	32 y	# series
AChE				X	Y	Y	X	X									Y	X		X				X		X	X		X	X		X	14
Iron																											X			X		X	3
Nissl	Y	X	X	X	X	X	X	X	X	X	X	X	X	X	X	Y	Y	X	X	X	X	Y	X	X	X	X	X	X	X	X	X	X	32
5‐HT	Y	Y		X		Y		X				X	X	X	X	Y	Y	X	X	X	X	Y	Y			X							18
a‐Syn (SNCA)																											X			X		X	3
b‐Amy (6E10)																											X			X		X	3
CB	Y	X	X	X	Y	Y	X	X	X	X	X	X	X	X	X	Y	Y	X	X	X	X	Y	Y	X		X		X	X				27
CCK	Y	Y		X								X	X	X	X				X	X	X	Y	Y			X		X					14
ChAT	X	X	X	X	X	X	X	X	X	X	X	X	X	X	X	X		X	X	X	X	X	X	X	X	X	X	X	X	X	X	X	31
CR	Y	Y	X	X	Y	Y	X	X	X	X	X	X	X	X	X	Y	Y		X	X	X	Y	Y	X		X		X					25
DBH											X	X									X												3
GABA				X																X							X						3
GABA‐B							X				X													X	X								4
GFAP																											X			X		X	3
Iba1	X	X	X	X	Y	X	X	X	X	X	X	X	X	X	X	X	Y	X	X	X	X	X	X	X		X		X	X	X		X	29
LTX				X																X							X						3
MBP	Y	X	X	X	Y	Y	X	X	X	X	X	X	X	X	X	Y	Y		X	X	X	Y	Y	X		X							24
NeuN	Y	Y	X	X	Y	Y	X	X	X	X	X	X	X	X	X	Y	Y		X	X	X	Y	Y	X		X	X	X	X	X	X	X	30
NOS	Y	Y		X	Y	Y		X				X	X	X	X	Y	Y		X	X	X	Y	Y			X			X				19
NPY	X	X	X	X	X	X	X	X	X	X	X	X	X	X	X	X	X	X	X	X	X	X	X	X	X	X	X	X	X	X	X	X	32
NRGN	Y	Y		X	Y	Y		X				X	X	X	X	Y	Y		X	X	X	Y	Y			X		X					19
Nurr1				X																X							X						3
Olig2	Y	X	X	X	Y	Y	X	X	X	X	X	X	X	X	X	Y	Y		X	X	X	Y	Y	X		X							24
PV	Y	Y	X	X	Y	Y	X	X	X	X	X	X	X	X	X	Y	Y		X	X	X	Y	Y	X		X	X	X	X	X	X	X	30
S100																												X					1
SATB2				X																X							X						3
SMI‐312		X	X	X			X		X	X		X			X		Y	X	X	X										X	X	X	15
SMI‐32	X	X	X	X	X	Y	X	X	X	X	X	X	X	X	X	X	Y	X	X	X	X	X	X	X		X	X	X	X	X	X	X	31
SOM	Y	Y	X	X	Y	Y	X	X	X	X	X	X	X	X	X	Y	Y		X	X	X	Y	Y	X		X		X					25
SP	Y	Y		X	Y	Y		X				X	X	X	X	Y		X	X	X	X	Y	Y			X							18
SV2a																											X			X		X	3
TauAT8																											X			X		X	3
TauP217																											X			X		X	3
TBR1				X																X							X						3
TH	Y	Y	X	X	Y	Y	X	X	X	X	X	X	X	X	X	Y	Y	X	X	X	X	Y	Y	X		X	X	X	X	X		X	30
# of Stains	4 (14)	9 (10)	14	25	4 (13)	4 (14)	16	18	14	14	15	20	18	18	19	4 (13)	1 (16)	11	19	25	19	4 (14)	5 (13)	15	4	19	20	14	11	17	7	17	424 (107)
# of sections	264	568	1027	1696	264	288	1106	1332	1008	1036	1080	1418	1282	1316	1292	284	71	765	1369	1840	1367	288	340	1154	312	1384	1524	1022	781	1292	504	1224	30,498
	924	630			858	1008										923	1136					1008	884										7371

*Note*: Table data shown only histology already done. Number of stains is shown as X(Y), where X = # already digitized and public, (Y) = # currently being digitized. Months and years are given to the closed digit; exact age can be obtained from the public series.

Abbreviations: F, female; M, male; m, months; P#, # of postnatal days; y, years.

### 3.2 Examples of ongoing research using MBRC materials

**FIGURE 1:**
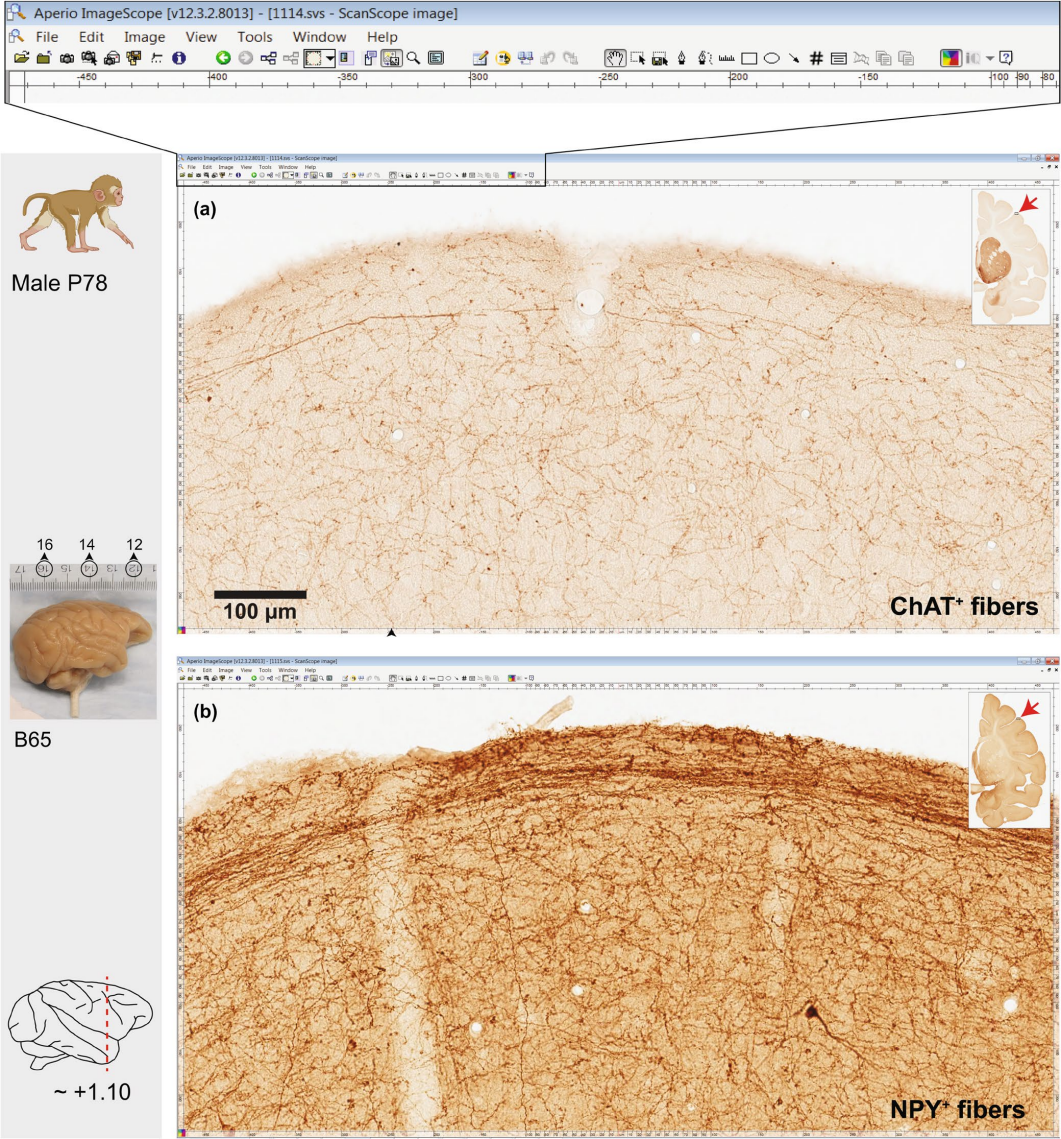
Cortical cholinergic (ChAT+) versus NPY+ axon terminals. We are systematically investigating differences in the density of ChAT+ fibers (a) and NPY+ fibers (b) in diverse cortical areas. We are collecting anatomical data to test the hypothesis that while cholinergic inputs serve a primary role to arouse the cortex, the NPY putative GABAergic cells provide the necessary inhibitory tone and that the distribution of axons from these two distinct cellular populations provide an important cortical physiological balance between excitation and inhibition. Here we illustrate the collection of raw data using the Aperio ImageScope viewing software. The top of the screen is magnified to make the icons visible. The left banner indicates these data are from a male 78 days postnatal (P78). A lateral view of the right hemisphere of the actual brain (B65) in MBRC Collection 6 is shown. Scale bar in A applies to B. Further details are in the text. For this and all subsequent figures, the left banner provides actual photographs of the brains or brain blocks used (if the photographs are available) and the diagram of the lateral view of the brain surface shows in mm the approximate rostro‐caudal coordinates (red vertical line) with respect to bregma (Paxinos et al., 2000).

**FIGURE 2:**
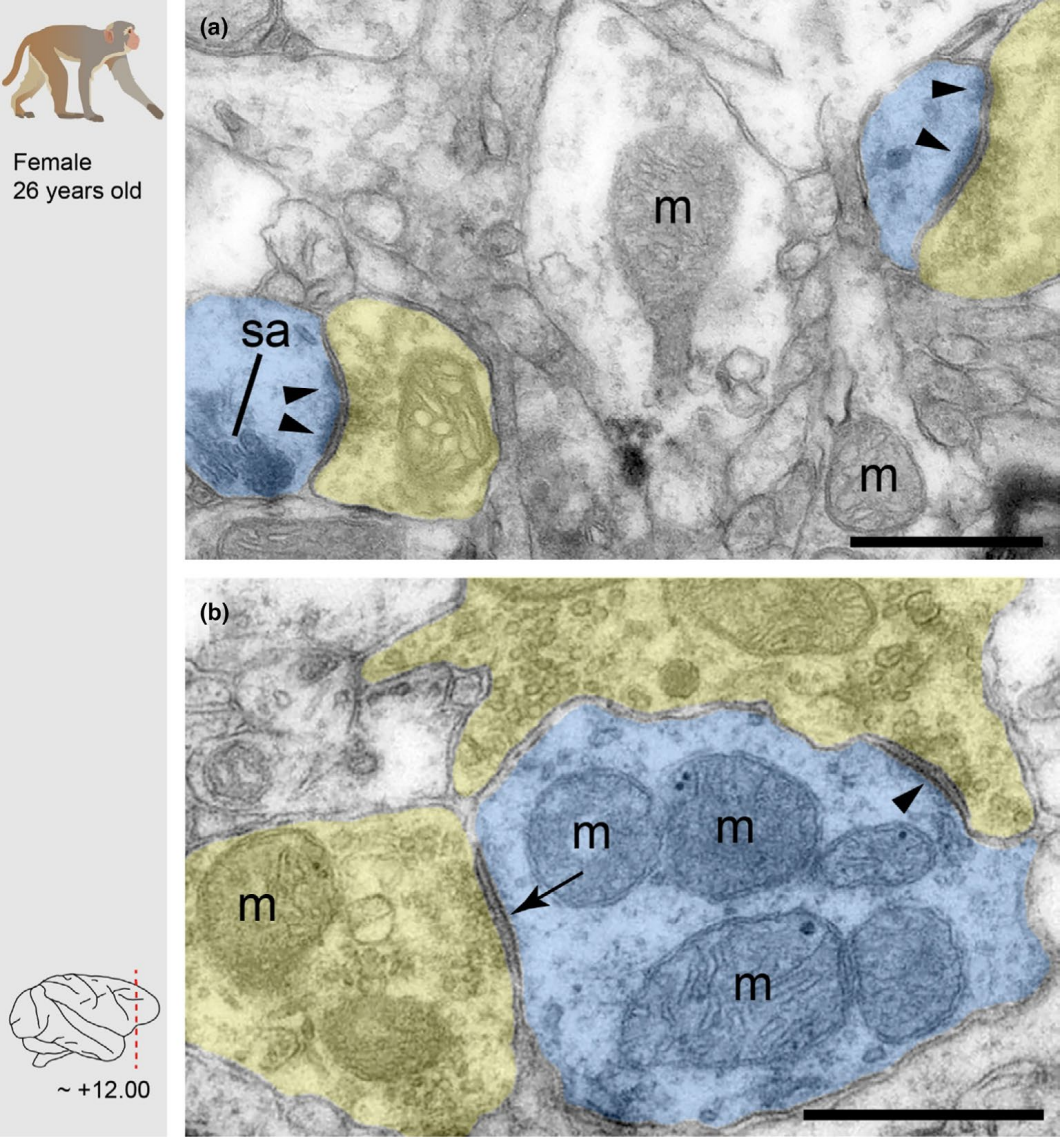
Electron micrographs of neuropil from 26‐year‐old Rhesus macaque neocortex layer 3. Synaptic boutons are highlighted yellow, postsynaptic dendrites—blue. (a) Two perforated asymmetric synapses (presumed glutamatergic; arrowheads) innervate dendritic spines identified with spine apparatus. (b) A dendritic shaft innervated by asymmetric (arrowhead) and symmetric (presumed GABA‐ergic; arrow) synapses. m, mitochondria; sa, spine apparatus. Scale bars, 0.5 μm. Materials from MBRC Collection 5.

**FIGURE 3:**
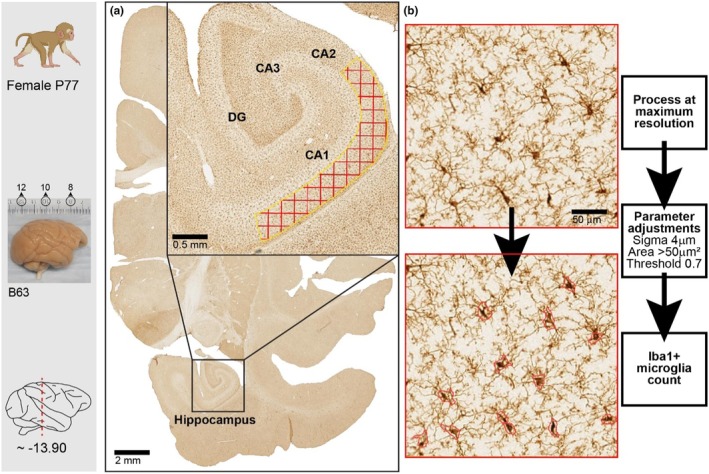
Iba1^+^ microglia quantification using QuPath in the CA1 region of the hippocampus. (a) Representative hippocampal section (B163 s37) and tiled sampling region used for cell quantification. (b) A high magnification image of Iba1^+^ microglia distribution within a 250 μm^2^ tile (section thickness 50 μm). QuPath automated positive cell detection is applied and optimized with maximum resolution processing and parameter adjustment. Similar processing has been done using ImageJ (not shown). This is a practical example of the use of new automated techniques for pattern recognition and cell counting. The left banner indicates these data are from a female 77 days postnatal (P77). Further details are in the text.

**FIGURE 4:**
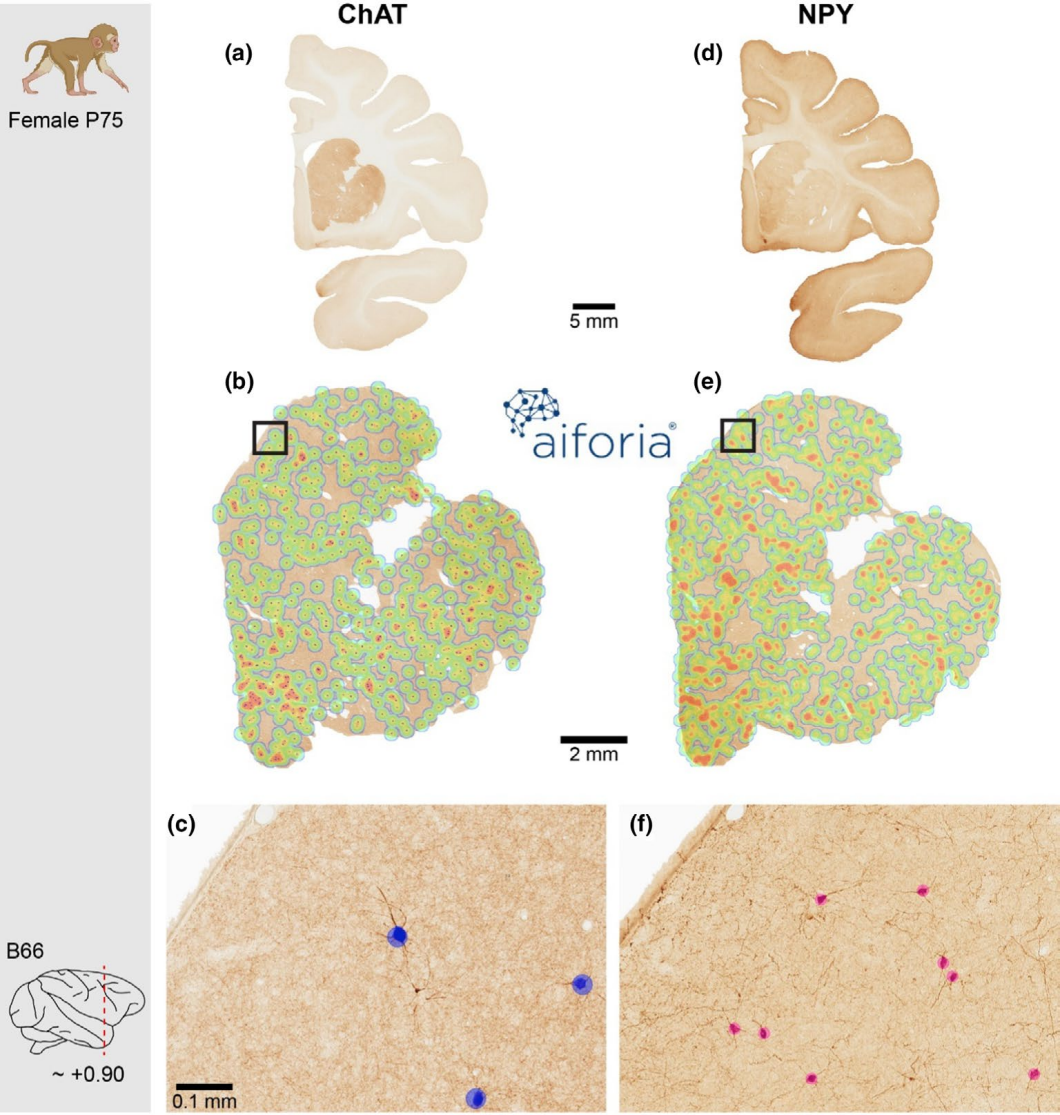
The density of NPY^+^ interneurons in the striatum is higher than that of ChAT^+^ interneurons. (a–d) Low magnification images of corresponding sections stained for ChAT (a) or NPY (D), from a 2.5‐month‐old female, B66 section 23 in both the ChAT and NPY series. (b–e) Detection of neurons using the AI platform Aiforia. The heat maps facilitate the distinction of differences in cellular density (red is high). (c–f) Illustrate 2 corresponding fields chosen at random. Notice difference in cell size and density between ChAT and NPY. Also, notice that at this level, ChAT density is higher in the ventral and medial quadrant and NPY density is higher in the medial zone than in the lateral. Scale bar in (c) applies to (f). Materials used are publicly available in MBRC Collection 6.

**FIGURE 5:**
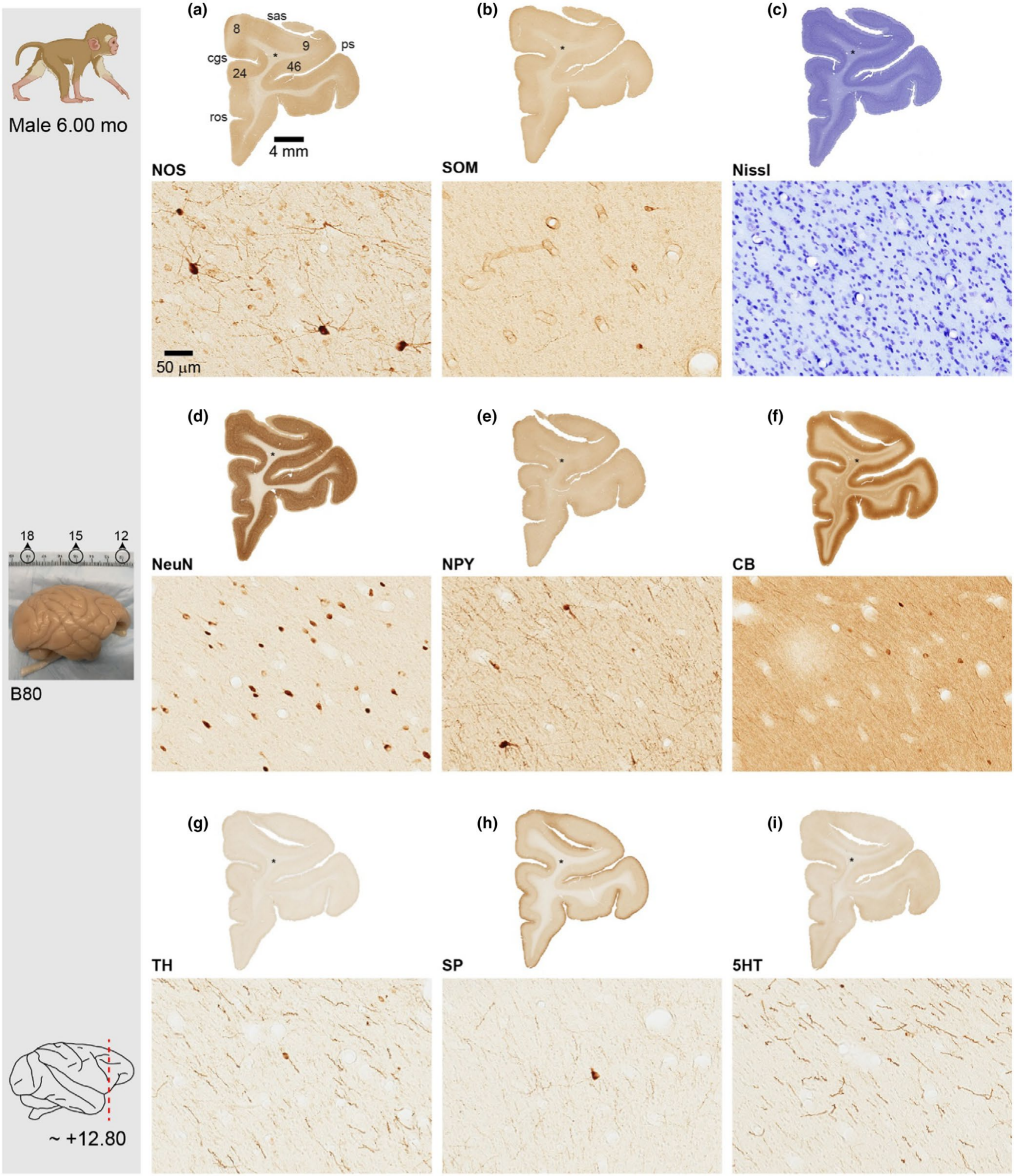
Neurochemical identity of white matter interstitial cells. (a–i) Low magnification panoramic views showing (a) NOS, (b) SOM, (c) Nissl, (d) NeuN, (e) NPY, (f) CB, (g) TH, (h) SP, (i) 5HT (see abbreviations in Table 1) in a 6‐month‐old M rhesus macaque (B80). Under each coronal section, a higher magnification view of the white matter content at the location marked by the asterisk is shown. Coronal sections are approximately 9 mm anterior to Bregma. The scale bars in (a) and in the NOS inset apply correspondingly to all panels. Labels in (a) are provided for orientation: Brodmann areas 8, 9, 24, and 46 approximately corresponding to frontal eye fields (8), dorsolateral prefrontal cortex (9), cingulate/limbic cortex (24), prefrontal cortex (9); ps, principal sulcus; cgs cingulate sulcus; sas, superior arcuate sulcus; ros, rostral sulcus; Materials used are publicly available in MBRC Collection 6.

**FIGURE 6:**
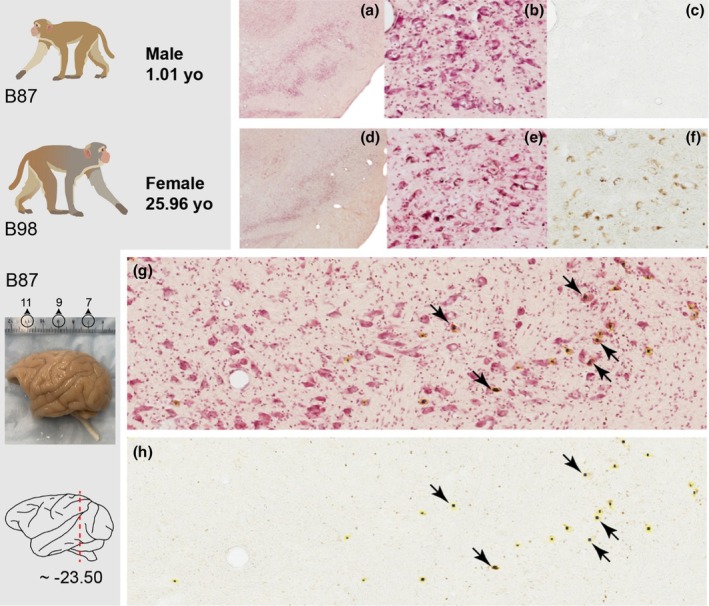
Age‐dependent neuromelanin accumulation in the Substantia Nigra (SN) of Rhesus Macaque. (a–c) SN of a young rhesus macaque (1.01 years). (a) Section stained with a red counterstain dye. (b) Higher magnification of the same region. (c) Same area without counterstain, representing the natural appearance of the tissue as well as the absence of detectable neuromelanin. (d–f) SN of an aged rhesus macaque (25.96 years). (d) Section stained with a red counterstain dye. (e) Higher magnification of the same region. (f) Same area without counterstain, where there is a high and intense amount of neuromelanin that appears as brown‐yellow granules. (g, h) Synchronized images of the same region in the aged monkey SN: (g) red counterstain and (h) natural appearance without counterstain. Arrows indicate the same dopaminergic neurons containing intracellular neuromelanin pigment. Materials processed from frozen tissue blocks in MBRC Collection 7.

**FIGURE 7:**
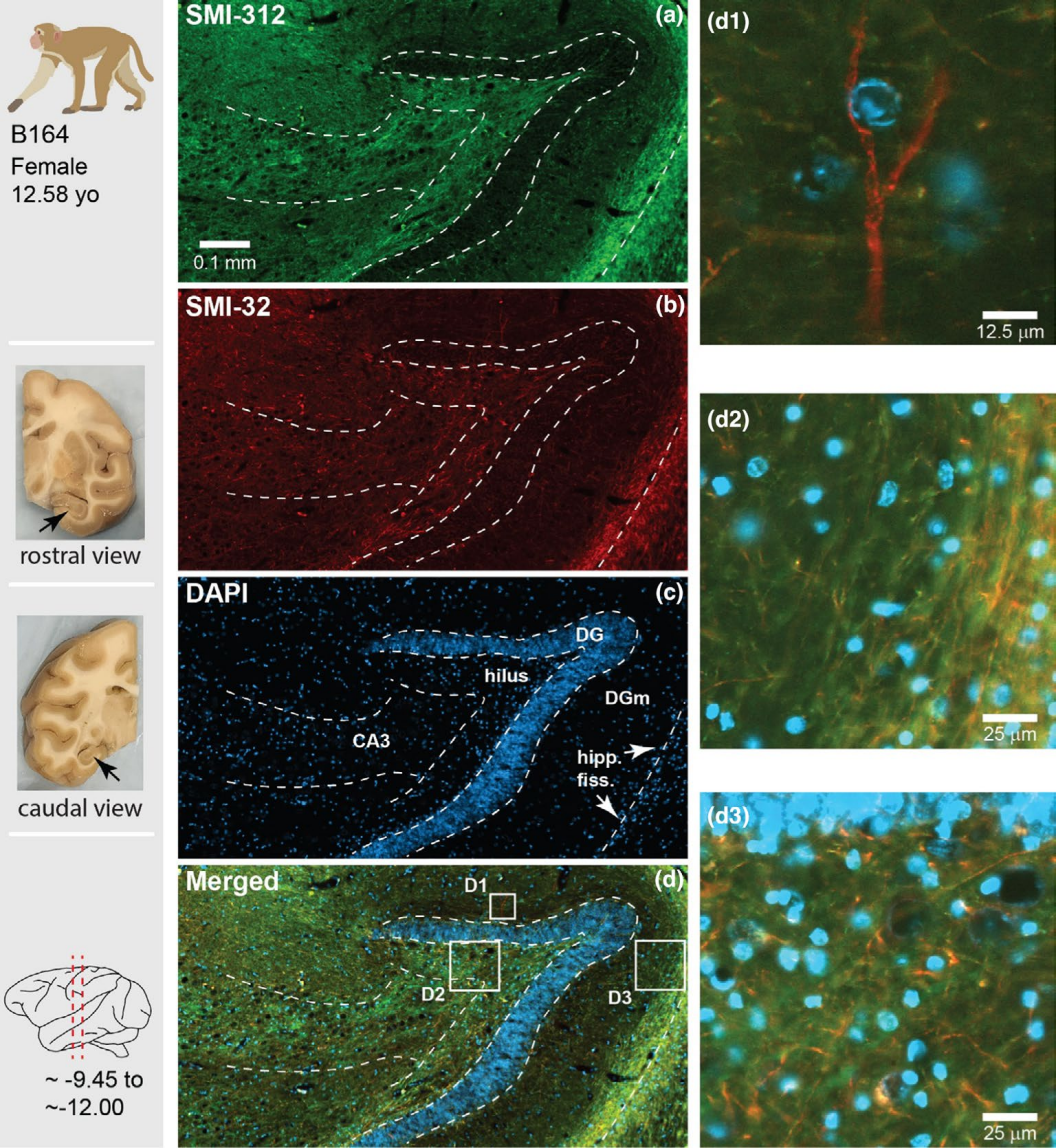
Co‐expression of phosphorylated and non‐phosphorylated neurofilaments in the adult NHP hippocampus. (a–d) Representative immunofluorescence images from a 12.5‐year‐old rhesus macaque, showing labeling for (a) phosphorylated NFs (SMI‐312^+^, green); (b) non‐phosphorylated neurofilaments (SMI‐32^+^, red); (c) DAPI (blue, nuclear DNA) provided for anatomical orientation; and (d) merged channels showing colocalization SMI‐312^+^ and SMI‐32^+^ (yellow/orange). (d1–d3) Corresponding higher magnification fields as indicated in (d). Notice that red fiber in (D1) is SMI‐32^+^/SMI‐312^−^ (red only). Scale bar in (a) applies to (b–d). Images were captured with a Zeiss Axioscan 7 digital scanner at 20×. Image analysis is done with Zen 3.10 lite. CA3, *cornu ammonis* field 3 of the hippocampus; DG, dentate gyrus; DGm, dentate gyrus molecular layer; hipp. fiss., hippocampal fissure. Materials processed from frozen tissue blocks available in Collection 7 of the MBRC.

**FIGURE 8:**
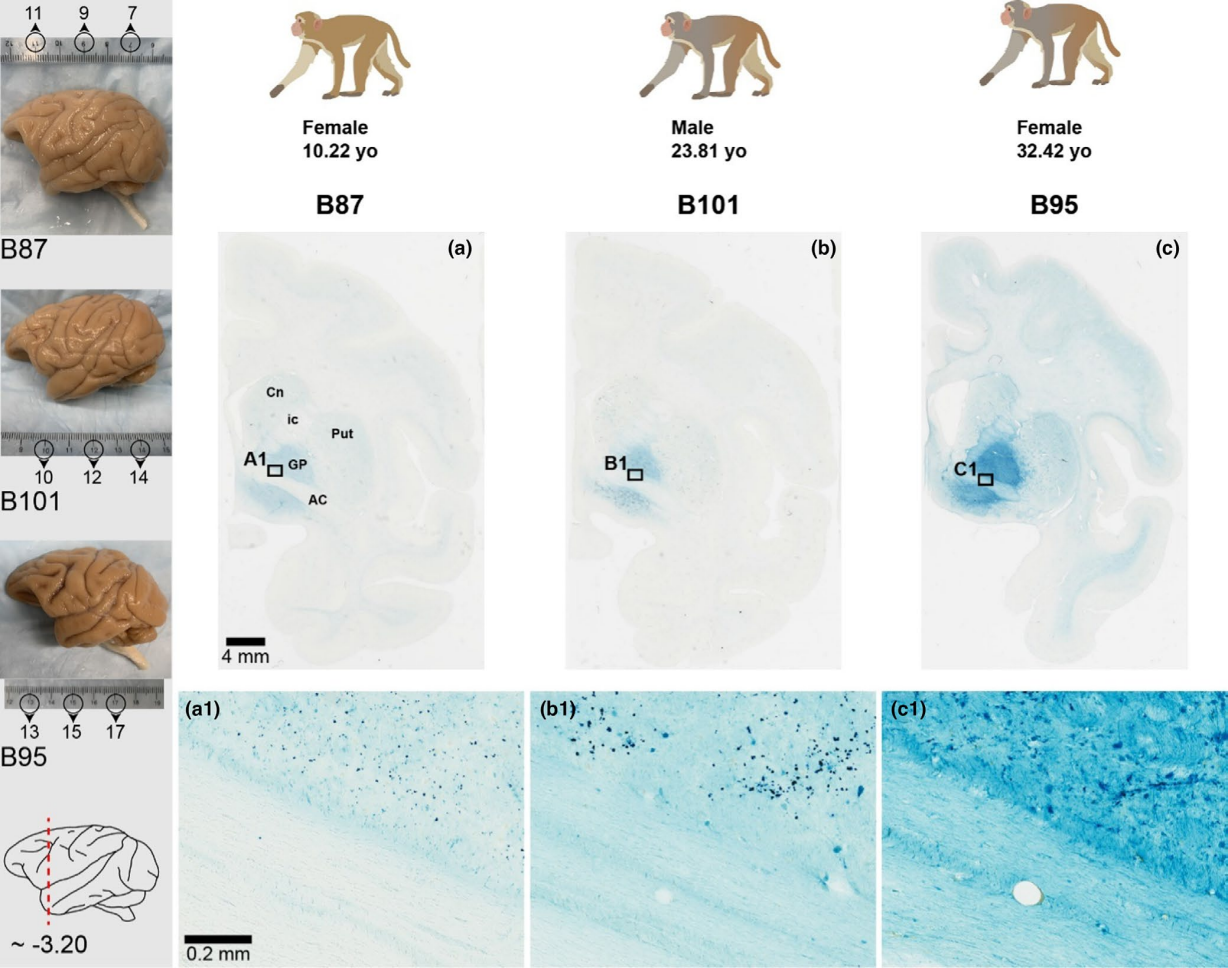
Age‐dependent iron accumulation in cortical and subcortical regions of the aging Rhesus Macaque. (a–c) Low magnification panoramic views showing iron accumulation as stained with the Prussian blue reaction in (a) a 10‐year‐old female (B88), (b) a 23‐year‐old male (B101), and (c) a 32‐year‐old female (B95). The age‐dependent changes in Prussian blue contrast is evident. Notice staining in cortical areas of the oldest specimen not observed in the younger ones. All the sampled brains are publicly available in MBRC Collection 6. Some further changes, including those related to AD, and that were investigated using MBRC materials are available in the literature (e.g., Zeiss, Duque, & Huttner, 2025; Zeiss, Huttner, et al., 2025). Scale bar and abbreviations in (a) apply to (b) and (c). Scale bar in (A1) applies to (B1) and (C1). Tissue available in Collection 7 was processed and the results incorporated to Collection 6. AC, anterior commissure; Cn, caudate nucleus; GP, globus pallidus; ic, internal capsule; Put, putamen.

The following figures are all from ongoing and unpublished research. They illustrate how we, and some of our colleagues, are using these materials to conduct de novo research. We emphasize the use of Collections 5, 6, and 7. Figure 1 illustrates an example of our systematic investigation of cholinergic and NPY axon terminals in different cortical areas. Here the samples are from the corresponding sections number 26 in each of the galleries (ChAT and NPY) in B65, a 78‐day‐old male monkey. The cortical area corresponds to the posterior granular prefrontal cortex, approximately Brodmann area 8 AD (the antero‐dorsal part of 8). This region is of interest, among other things, because it is believed to be involved in top‐down cognitive control. In this study, we are testing the general hypothesis that cortical cholinergic inputs serve a primary role to arouse the cortex while the NPY putative GABAergic cells provide inhibitory tone and that their physiological strength is reflected in the number of structures (axons) that subserve these functions. We are in the process of measuring and quantifying differences in cholinergic and NPY terminals in different territories of the rhesus macaque prefrontal cortex. The main tool for this study is the Aperio ImageScope program. Figure 2 shows and example from Collection 5. This female was case number 101212 (i.e., sacrificed October 12, 2012) and it samples layer 3 of the prefrontal cortex using electron microscopy. This is part of data collection for the study described for Figure 1. Figure 3 is from a study titled “Spatial statistics and point pattern analysis reveal lifespan trajectories of microglial density and clustering in the primate hippocampus” by our collaborators Aya Nusir and Dr. Nadine Kabbani. The study is now under review in Scientific Reports. In this study, 15 female rhesus macaques spanning late gestation to elderly, all in Collection 6, were used to quantify microglia density and spatial patterning in the hippocampus. The findings reveal that hippocampal microglia undergo subregion‐specific remodeling across the female primate lifespan. Several different techniques have been used for analysis most prominently QuPath and ImageJ. Additional studies on this subject using MacBrain materials are ongoing. Figure 4 illustrates two MBRC initiated studies in the basal ganglia, one on cholinergic interneurons and the other on NPY interneurons. In short, we are looking into the genesis, detection of neurochemical identity, and age‐related changes in these interneuron populations within the striatum. Our interest is to understand how striatal cell numbers and distributions vary throughout life as an essential step to our understanding of their involvement in normal and pathological physiology. These studies are not in isolation but will be coupled to other studies we have initiated in cortex and thalamus. We are testing a hypothesis of circuit deterioration in hierarchical order. The striatum was chosen to be explored by the artificial intelligence platform Aiforia because as a region of interest it is relatively easy to identify and delimit in different staining conditions. Figure 5 illustrates ongoing studies on interstitial white matter neurons for which we have already provided some results (see for instance Ahmed et al., 2024). In this study with our colleagues and collaborators Dr. Bashir Ahmed and Dr. Zoltan Molnár, we are exploring the numbers, distributions, and neurochemical identity of interstitial cells in different cortical regions; here we show NOS, SOM, Nissl, NeuN, NPY, CB, TH, SP, and 5HT at the level of the prefrontal cortex (PFC). Data collection and analysis are carried out in parallel using QuPath in addition and Stereo Investigator. Figure 6 illustrates ongoing studies related to PD in a collaboration with Dr. Jose L. Lanciego. The materials processed by our colleagues Julia Chocarro and Cristina Canales while at the MBRC were obtained from tissue blocks available in Collection 7. The figure illustrates the investigation of age‐dependent neuromelanin accumulation in the Substantia Nigra (SN) of Rhesus Macaque and the study is extending into the locus coeruleus and dorsal nucleus of the vagus nerve. For this investigation, the primary tool being used is our AI platform Aiforia. Figure 7, also prepared from tissue blocks in MBRC Collection 7 is expanding our previous studies (e.g., in the amygdala see: McDonald & Duque, 2022) of the expression of phosphorylated (SMI‐312^+^) and non‐phosphorylated neurofilaments (SMI‐32^+^) in different brain structures. This study, still in the data collection phase, is testing the hypothesis of region‐specific age dependent changes in the expression of phosphorylated vs. non‐phosphorylated neurofilaments in the hippocampus. Initial data analysis of fluorescent materials is being done with Zeiss ZEN software, an AI‐driven microscopy platform we use for image processing and analysis after image acquisition from our Zeiss Axioscan 7 scanner. QuPath and ImageJ are also being tested for specific parts of this project. Figure 8 illustrates age‐dependent iron accumulation in cortical and subcortical regions in studies relevant to AD. The materials used encompass Collections 6, 7, and 8. Data collection and analysis is a continuation of work done in cooperation with Yale's Alzheimer's Disease Research Center (ADRC). For further details, see for instance (Zeiss, Duque, & Huttner 2025; Zeiss, Huttner, et al., 2025).

## 4 DISCUSSION

This discussion consists of 3 parts. (1) We present a summary of the contents of some other publicly available datasets, which we identified to be the richest in terms of relevant data for comparison to that available in the MBRC. (2) With a global view, we stress what is available in the MBRC, which is not found, limited, or is additional to what those other datasets contain. (3) We lay down the foundations of our plans to expand the Collections in the MBRC over time.

This, we hope, will highlight the limitations, strengths, and complementarities of the different datasets which, together, can provide neuroscientists with a richer repertoire of tools to use the rhesus macaque NHP model in their studies. We trust in the power of sharing and collaboration, and it is our firm belief that only through better utilization of resources, archiving materials and collective efforts, researchers in the field will be able to adopt new and emerging technologies that can truly contribute to a decrease in animal sacrifice.

### 4.1 Summary of NHP datasets

BrainMaps.org, was developed by the University of California, Davis. This database is a high‐resolution digital neurohistology repository with 165 datasets across 24 documented species. Among the species registered, *Macaca mulatta* appears as the most extensively represented, accounting for 31 datasets as of mid‐2025. These datasets provide metadata on staining methods, anatomical regions, sectioning planes, image resolution, and histological preparation. Most *M. mulatta* datasets are coronally sectioned (25 out of 31), with the remainder divided across sagittal (2), horizontal (2), and multiple planes (2). Staining protocols include Nissl (13 datasets), Parvalbumin (3), Calbindin (1), Calretinin (1), Cytochrome Oxidase (1), non‐phosphorylated neurofilament H (1), Cholera Toxin subunit b (4), and potassium channel markers such as Kv2.1. Weil and non‐stained anatomical photography are also represented. Resolutions for *M. mulatta* datasets range from 0.46 to 300 μm/pixel, with section thicknesses from 25 to 400 μm and slide counts between 1 and 507. Across the whole repository, image resolutions span from approximately 0.0005 to 860 μm/pixel depending on modality, and some datasets contain up to 1975 slides. Imaging modalities include brightfield microscopy, immunohistochemistry, anterograde and retrograde tracer studies, MRI, and EM. While metadata on specimen sex and strain are rarely provided and not standardized across datasets, core variables such as staining method, anatomical region, section orientation, and technical specifications are, for the most part, consistently available. All images are accessible through an interactive, zoomable web interface that allows detailed examination of high‐resolution brain sections. Although the platform does not support bulk downloads or integration with external analysis pipelines, its consistent metadata structure, broad taxonomic coverage, and high anatomical resolution make BrainMaps.org a valuable and well documented resource for digital comparative neuroanatomy, especially for manual exploration and reference across species.

BrainCatalog is a digital neuroanatomy repository developed through a collaboration between the Institut Pasteur, the Jardin des Plantes, and the Institut du Cerveau et de la Moelle (ICM), with additional support from the Natural History Museum in Paris and the Cogimage team at ICM. As of mid‐2025, manual tally indicates MRI anatomical datasets from approximately 37 species spanning primates, carnivores, ungulates, marsupials, and monotremes. Each species is represented by a single high‐resolution MRI scan that can be segmented into sagittal, coronal, and axial planes, depending on the specimen, with slice counts varying across species. In the case of *Macaca mulatta*, the images include 182 sagittal, 234 coronal, and 147 axial slices. The datasets capture structural details of brain regions such as the telencephalon, hippocampus, striatum, cerebellum, corpus callosum, and neocortex, but the resolution values are not disclosed. The repository does not provide histological data or specimen metadata such as sex, age, or strain, and tissue preparation protocols are not applicable given its exclusive reliance on MRI. While the anatomical segmentations indicate regional labeling, there is no integration with dynamic atlas tools or coordinate systems. Data are accessible online under a Creative Commons license, though only as navigable image slices without bulk download options or programmatic interoperability. However, 3D surface reconstructions are available for some specimens as individual downloadable files in PLY format, corresponding to overall brain surfaces and each segmentation plane (sagittal, coronal, axial). Technical documentation is minimal, and no persistent identifiers or user support channels are provided. Despite these limitations, the Brain Catalogue offers a comparative view of brain morphology across a wide range of species, supporting research in digital comparative neuroanatomy.

The Digital Brain Zoo, part of the Digital Brain Bank maintained by the Wellcome Centre for Integrative Neuroimaging at the University of Oxford and supported by the Wellcome Trust and the UK Medical Research Council, provides postmortem MRI datasets across multiple non‐human mammals for comparative and evolutionary neuroscience. It features complete brain structural and diffusion imaging data acquired at resolutions between 300 and 600 μm using ultra high field scanners. As of mid‐2025, the collection includes 14 NHP species representing all major branches of the primate lineage—including *Macaca mulatta*, chimpanzee, gorilla, and tamarin, alongside species from Carnivora, Marsupialia, and Cetacea. The rhesus Macaque preview dataset includes a structural template and dyadic orientation maps created by combining and averaging data from three individual macaque brains. These can be explored using Tview, a custom web‐based viewer supporting multiscale visualization. For this species, Tview displays sagittal, axial, and coronal slices, with the top row showing the reference image without diffusion contrast and the bottom row representing the direction of water diffusion (red = left to right, blue = top to bottom, green = front to back). The full dataset includes preprocessed outputs such as Tensor, Ball, and Sticks models. Although information on age and sex is incomplete, this database emphasizes broad phylogenetic representation over complete demographic annotation. The Digital Brain Zoo enables easy comparison of brain structure across different species.

The SC21 Subcortical Digital Atlas is a high‐resolution atlas of subcortical brain regions in *M. mulatta* that complements and extends the earlier D99 cortical atlases of Saleem and colleagues (Reveley et al., 2017; Saleem et al., 2021; Saleem & Logothetis, 2012). This atlas was developed at The Center for Neuroscience and Regenerative Medicine (CNRM) of the Uniformed Services University (USU), in partnership with several NIH institutes. Methodologically, it was created by integrating high‐resolution Mean Apparent Propagator—Magnetic Resonance Imaging (MAP‐MRI) techniques with detailed histological analysis from a single adult male rhesus macaque. This multimodal resource combines ex vivo 7T MRI data with five histological stains (Nissl, AChE, SMI‐32, Parvalbumin, and ChAT), enabling the anatomical identification of 219 subcortical regions in total. This includes the segmentation of 190 gray matter subregions and 29 fiber tracts. The brain was coronally sectioned at 50 μm thickness, yielding 1420 sections across 10 series, five of which were processed for histology. MRI acquisition included MAP‐MRI at 200 μm isotropic resolution and T2‐weighted and magnetization transfer imaging. Microstructural features were evaluated using standard diffusion tensor measures: mean diffusivity (MD), fractional anisotropy (FA), axial diffusivity (AD), and radial diffusivity (RD). Orientation and anisotropy were described with directionally encoded fiber orientation maps (DEC‐FOD) and propagator anisotropy (PA). Additional MAP‐MRI parameters included non‐Gaussianity (NG), return‐to‐origin probability (RTOP), return‐to‐axis probability (RTAP), and return‐to‐plane probability (RTPP). These MRI volumes were aligned with the stained sections to support manual segmentation. The SC21 volume was registered to the D99 cortical template, producing the updated D99 Version 2.0 atlas that uses both cortical and subcortical parcellations in a 3D space. The dataset is available in NIfTI and GIFTI and is compatible with neuroimaging platforms like AFNI (Analysis of Functional NeuroImages) and SUMA (Surface Mapper). Although the atlas is based on a single individual and lacks metadata, its anatomical precision, multimodal integration, and compatibility with macaque brain workflows makes it a helpful tool for structural mapping, deep brain targeting, and neuroanatomical research. The atlas can be freely downloaded as a single compressed file (tarball).

Brainmuseum.org is a project developed through collaboration between the University of Wisconsin, the Michigan State Comparative Mammalian Brain Collections, and the National Museum of Health and Medicine. It provides online access to photographs and histological sections from over 100 mammalian species, representing more than 270 brain specimens across over 20 mammalian orders. Most species are represented by whole‐brain photographs taken from multiple perspectives. As of mid‐2025, a manual tally identified 64 species with brain section atlases available under the ‘Brain Sections’ tab. These brain sections were stained using thionine (to highlight somata), hematoxylin (to visualize nerve fibers), or both. In some cases, such as the chimpanzee, additional images focus on specific brain regions like the thalamus. A key limitation is that most species are represented by only one individual, and sex and age information are inconsistently reported, limiting inter‐ and intraspecies comparisons.

Brains were prepared by in situ perfusion with 10% saline followed by formol‐saline. They were embedded in celloidin mixtures of increasing viscosity and sectioned serially at 25–40 μm using a sliding microtome. While most brains were sectioned coronally, a few were cut along horizontal or sagittal planes, with slight adjustments based on brain age and size.

For the rhesus macaque, the site provides one completely sectioned coronal brain along with species‐specific data such as gestation time, geographic range, and behavioral characteristics. Two whole‐brain images are available—one showing six standard views and another showing a left dorsolateral view. There are images for a total of 104 thionine‐stained sections although no higher magnification images or fiber stains are available. Data for other primates include humans, chimpanzees, three‐striped night monkeys, mandrills, mongoose lemurs, squirrel monkeys, grey mouse lemurs, white‐headed capuchins, northern plains gray langurs, pottos, southern pig‐tailed macaques, mantled howler monkeys, and red‐bellied titi monkeys. Additional primate species are included with whole‐brain images only (i.e., without images of brain sections).

The Blueprint Non‐Human Primate (NHP) Atlas provides gene expression and neuroanatomical data for the rhesus macaque brain. The site is organized into modules for Microarray (microdissection), In Situ Hybridization (ISH), and Reference Data (https://www.blueprintnhpatlas.org/static/referencedata). This last one uses MRI and Nissl histology categorized by developmental timepoints, supporting integration with the microarray and ISH datasets. This project is part of the interconnected products of the Allen Brain Institute, and hence, Brainevolution.org and AtlasBrain‐map.org have links to the NIH Blueprint Non‐Human Primate (NHP) Atlas. The site for Brainevolution was developed by the Allen Discovery Center for Human Brain Evolution at Boston Children's Hospital and Harvard Medical School. It includes the Ancient DNA Resource, the UCSC Genome Browser, and tools for analyzing neuronal circuitry. The resources and tools the Allen Institute offers include Microdissections to study fine structure transcriptional profiles across prenatal and postnatal development, and Macrodissections to study gross structural transcriptional profiles across postnatal development, in addition to cellular resolution in situ hybridization (ISH) data. There are over 10 cortical areas, hippocampus and dorsal lateral geniculate nucleus obtained using laser microdissection with each area assayed in duplicate in both M and F adult animals using Affymetrix GeneChip Rhesus Macaque Genome Arrays (Home:: NIH Blueprint Non‐Human Primate (NHP) Atlas). The atlases can be downloaded, and Supplemental Data include MRI zip files containing DICOM used for the ISH. DNA microarray data can be searched by topic.

At the time of writing, the Atlas includes macaques from embryonic ages: E40, E50, E70, E80, E90, and E120. For ages E40 to E90, images stained for Nissl, ENC1, and Gap43 are available. At E120, staining is available for Nissl and AChE. Each embryonic age includes samples from four monkeys, 2 M and 2 F. The site also provides data for M specimens at four postnatal ages: 0, 3, 12, and 48 months. These stages include gross structural transcriptional profiling files in zip format, MRI imaging, and section images stained with Nissl across both hemispheres. The number of section images ranges from approximately 60 in early embryonic stages to over 200 in later embryonic and postnatal ages.

The site features a high‐resolution image viewer tool, which allows users to examine and compare two sections side‐by‐side. While the viewer lets you browse sections, data packages are available for download via the Download tab. Further information on the database can be found in the Allen Institute Brain Map Community Forum (https://community.brain‐map.org/). A section dedicated to the NHP databases is available at https://community.brain‐map.org/c/how‐to/nhp‐atlas/30, and detailed documentation and technical white papers can be accessed through https://community.brain‐map.org/t/documentation‐for‐nhp‐atlas/3075.

The Scalable Brain Atlas (SBA) is an online platform developed by Rembrandt Bakker, initially under the supervision of Rolf Kötter at Radboud University Nijmegen, with continued support from the International Neuroinformatics Coordinating Facility (INCF). SBA functions as a portal for published digital brain atlases. Rather than producing primary data, SBA organizes templates from contributing research groups and distributes them through a standardized framework for visualization, comparative analysis, and integration with external Neuroinformatics resources.

As of mid‐2025, the SBA has at least four rhesus macaque atlases that differ in methodology and anatomical coverage. The Calabrese et al. template is a diffusion tensor imaging (DTI) and MRI atlas built from 10 postmortem macaques scanned at 7T, with isotropic voxel sizes of 0.075 mm for structural MRI and 0.15 mm for DTI (Calabrese et al., 2015). This atlas includes 241 anatomical labels adapted from Paxinos et al. (2000) and Puelles et al. (2013) and is archived at the Duke Center for In Vivo Microscopy. SBA provides downsampled versions of these data. The Paxinos atlas contributes stereotaxic delineations of major regions including the cortex, amygdala, thalamus, and striatum. These contours, first published in The Rhesus Monkey Brain in Stereotaxic Coordinates (Paxinos et al., 2000), were later integrated into 3D visualization tools such as the CoCoMac‐Paxinos viewer and remain accessible through SBA. The NeuroMaps atlas is derived from an ex vivo MRI of a 3‐year‐old male rhesus macaque scanned at 0.15 mm isotropic resolution on a 4.7T Bruker system (Paxinos et al., 2000). It includes 486 coronal, 346 horizontal, and 191 sagittal sections (50 μm thick), with up to 90 annotated structures per section. The parcellations cover cortical, subcortical, brainstem, and cerebellar regions. Finally, the Markov et al. (2014) atlas is restricted to neocortical parcellation, defining 91 cortical areas based on architectonic and connectivity data. It underlies the weighted and directed 29 × 91 connectivity matrix derived from more than 1600 retrograde tracer injections. SBA distributes atlas data in multiple formats, including SVG coronal sections, NIfTI volumes or 3D meshes, with connectivity matrices available through Core‐Nets.org (Markov et al., 2014). Scope and resolution can vary, and most resources are derived from a limited number of individuals, often a single specimen. Metadata such as specimen sex or age are rarely provided. Supporting Information and methodological details are primarily found in the original publications, while SBA provides .x3d files for interactive visualization via its web portal (https://scalablebrainatlas.incf.org/index.php).

Ebrains.eu provides atlases for humans, macaque monkey, rat, and mouse brains. An interactive 3D anatomical atlas for macaque monkey employs siibra‐explorer, an intuitive viewer which stands for “software interfaces for interacting with brain atlases.” Four views of the brain are shown simultaneously: coronal, sagittal, dorsal, as well as an axonometric cut out. Visitors to the site can slide cutting planes forward/backward and up/down to move progressively through brain levels. Brain regions are parcellated and labeled in different colors. There is a search bar to target specific brain regions, and the current coordinates are noted on the screen. The coordinates can be saved in the clipboard and noted for future reference.

The Macaque atlas is composed from the MEBRAINS Template and the Julich Brain Macaque Maps. The MEBRAINS template is population based, representing an average of high‐resolution structural T1 and T2 MRI scans as well as CT. They were done on 10 animals, 7M and 3F young adult rhesus macaques with an average age of 5.30 years. The Julich Brain Macaque Maps provide delineations of cyto‐ and receptor architectonically identified areas, based on the densities of 14 different receptors for multiple classical neurotransmitters. Data come from one adult *M. Mulatta* (sex not stated) and three adults male *M. fascicularis*. The site excels at providing insight into the 3D organization of brain anatomy, but the zoom resolution is relatively low since it is based on MRI and CT scans. See: https://www.ebrains.eu/brain‐atlases/reference‐atlases/monkey‐brain/ and MEBRAINS Multilevel Macaque Brain Atlas—Atlas composition, MEBRAINS + Julich Brain (Balan et al., 2024). Interactive viewer is available at: https://atlases.ebrains.eu/viewer/#/a:juelich:iav:atlas:v1.0.0:monkey/t:minds:core:referencespace:v1.0.0:MEBRAINS/p:minds:core:parcellationatlas:v1.0.0:e3235c039c6f54c3ba151568c829f117/@:0.0.0.‐W000.._eCwg.2‐FUe3._‐s_W.2_evlu..3gfU..mr0~.VHz0~.BSR0..16SE/vs:v2‐ff011b03.

The PRIMatE Data Exchange (PRIME‐DE) is part of the International Neuroimaging Data‐sharing Initiative (INDI). It is hosted on the Neuroimaging Informatics Tools and Resources Clearinghouse (NITRC), which provides the infrastructure for open neuroimaging data. PRIME‐DE began in 2018. The initial release of PRIME‐DE included 25 collections from 22 sites, including data from 217 NHPs (Milham et al., 2018). The catalogue has since grown to include large rhesus macaque cohorts, most prominently a *M. mulatta* dataset with 592 individuals from the University of Wisconsin–Madison, in addition to other rhesus and cynomolgus macaque datasets. The NIN Primate Brain Bank contributes further by including datasets from strepsirrhines, New World and Old‐World monkeys, great apes, marmoset, baboon, chimpanzee, and gorilla. The repository offers structural T1 and T2 weighted MRI, functional MRI acquired during resting state and task performance, and diffusion MRI. Data have been collected on scanners ranging from 3T to 7T under awake and anaesthetized conditions, with a smaller subset of postmortem imaging also available. Histological data are not included in the resource. Metadata changes in different collections but commonly include basic demographic variables such as age and sex, along with acquisition parameters. Imaging quality is systematically assessed using the Preprocessed Connectome Project Quality Assurance Protocol (QAP). Access to PRIME‐DE requires free registration on NITRC.

A complementary resource present in PRIME‐DE is the Primate Resource Exchange (PRIME‐RE), which complements PRIME‐DE by providing standardized rhesus macaque templates and atlases (NMT, D99, CHARM, SARM, RheMAP warps). PRIME‐RE also includes analysis pipelines, protocols, and community forums (Messinger et al., 2021). The NIMH Macaque Templates (NMT v1.3 and v2.0) provide volumetric references generated by nonlinear averaging of T1 weighted scans, with version 2.0 offering both symmetric and asymmetric templates, improved brain masking, and stereotaxic alignment (Jung et al., 2021; Seidlitz et al., 2018). Integrated within NMT v2.0, the Cortical Hierarchy Atlas of the Rhesus Macaque (CHARM) subdivides the cortical sheet into six hierarchical levels, ranging from broad lobe divisions to fine‐grained parcellations based on the D99 atlas, thereby supporting analyses at multiple spatial scales. The Subcortical Atlas of the Rhesus Macaque (SARM) provides a corresponding hierarchical parcellation of the subcortex, designed for both structural and functional MRI studies (Hartig et al., 2021). The D99 atlas itself builds on an MRI and histology framework, with version 2 offering refined segmentation of cortical and subcortical structures in a single 3D volume (Reveley et al., 2017; Saleem et al., 2021). These resources establish a common coordinate framework that enables reproducible analyses and comparisons across studies in NHP neuroimaging.

The CocoMac 2 database is maintained by the Computation and Systems Neuroscience group at the Juelich Research Institute. The purpose of the database is to act as a repository and search tool for results from neuronal tract‐tracing experiments. This search tool allows users to identify brain regions and then receive spreadsheet data showing other brain regions where other studies have reported axonal connectivity. One of the database's key functions is to clarify inconsistencies in brain region labeling and definitions to ensure the accuracy of comparisons between studies. This source is limited to publications on tract‐tracing experiments in rhesus macaques but does not include histological data, images, staining information, or technical metadata, as those details remain in the original papers. Data can be viewed online and may be exportable as connectivity tables and matrices. SQL query capabilities support effective searching and may support meta‐analyses. This database does not seem to be updated very frequently. An original version CocoMac 1, is stated to have included over 450+ publications in its data repository, however the website states that the new and prior version are not currently merged, but that integration is ongoing (Bakker et al., 2011). The CoCoMac repository is also maintained by the German Neuroinformatics Node. In addition, CoCoMac is integrated with the Scalable Brain Atlas, which allows users to visualize connectivity data directly on brain maps.

*The BALSA* repository maintained by The Washington University in St. Louis has two main purposes: (1) to house user‐submitted neuroimaging data associated with published figures and (2) reference data mapped to brain atlas surfaces and volumes in humans and NHPs. At the time of this report, the repository listed 18 Rhesus monkey neuroimaging data sets along with other species such as humans and chimpanzees. Neuroimaging data are obtained using MRI/fMRI. Details related to these and other data sets (e.g., age, sex, treatment, brain regions), if available, can be accessed through the published articles and following links provided with each data set. Researchers can also access upcoming changes and future related uploads. To access new releases, they need to register and submit requests, and restricted data will be accessible to those who get approved. BALSA currently does not provide any cellular or molecular level research material from Rhesus monkeys but, in their “Perspective for NHP_NNP” study, they state that they will combine histological and imaging methods.

There are other data sets, many of which contain other species of importance for comparisons to NHP. Of these, we consider those containing human materials to be among the most relevant. A lot of those data sets contain invaluable imaging, histological, genetic, developmental, and pathological data, useful for guiding research and interpretation of what can be done with NHPs, the synergistic interplay between NHP data and human data will improve NHP models. Of the many datasets, we highlight those arising from the human connectome project, for further information see: HumanConnectome.org.

### 4.2 Global comparison to the materials in the MBRC collections

In general, many of the available datasets contain imaging, primarily MRI, with and without registration attempts to actual histology. Some are starting to add proteomics, gene expression and DNA microarray profiles, ISH data and so on. There is variability in terms of data from M and F, different ages, planes of orientation, and whether the data comes from one or both hemispheres. In all cases, there are limitations, and no dataset is considered to be complete. Older data is oftentimes missing sex and age details. Although the datasets so far available in the Collections of the MBRC are also limited and while currently lacking some data categories, such as MRI and gene expression mapping, they are the richest in terms of available histology. MBRC Collections 1 and 5 are unique and examples of ^3^H‐TdR materials at sequential developmental ages were not found anywhere else. Similarly, EM blocks available for research were also not found. These 2 Collections encompass more than *n* = 100 different M and F specimens from embryonic to adult. The lack of these types of data in the reviewed datasets indicates a serious gap in newer data acquisition and highlights the importance of the availability of these materials in the MBRC. EM, for instance is nowadays tagged as an old technique yet, there is no modern analogue technique capable of producing anatomical data at EM resolution. A similar situation applies to materials in Collection 2 (tract tracing injections), Collection 3 (prenatal and postnatal lesions) and 4 (prenatal X‐ray irradiation). The closest to Collection 2 is The CocoMac 2 database but, instead of actual materials a reference to many studies is provided.

In terms of materials in Collection 6 (https://macbraingallery.yale.edu/collection6/), in comparison for instance to BrainMaps.org, the MBRC has not yet published histo and immunohistological data in the sagittal and horizontal planes. However, such data are currently being processed and will become available in the future. Although the number of available specimens and stains is by far higher in MBRC Collection 6 than in any other of the databases explored, some datasets do have extraordinarily valuable and rich materials. For instance, BrainMaps has Cytochrome Oxidase, Cholera Toxin subunit b and potassium channel markers KChIP2b and Kv2.1 which are not yet available in the MBRC materials. In comparison to the Blueprint Non‐Human Primate (NHP) Atlas and other superb materials available through the Allen Brain Institute, embryonic data already processed in the MBRC promises to be richer in terms of the number of different stains (> 30 so far processed: 5‐HT, AChE, BIII‐Tubulin, CB, CCK, ChAT, CR, DCX, Fibronectin, GFAP, Iba1, Ki67, MBP, NCAN, NeuN, Nissl, NOS, NPY, NRGN, Olig‐2, PAS‐Alcian, PCNA, PV, Reelin, S100, SMI‐312, SMI‐32, SNAP25, SOM, SOX2, SOX5, SP, Synaptophysin, TBR1, TBR2, TH, and Vimentin) that will after further quality controls be released to the public. The embryonic ages currently in preparation include 24 specimens with both M and F and span E40 to E150. Sections are coronal and 50 microns thick but not all of the different stains are available at all embryonic ages. Also, similar to Allen Brain Institute materials, MBRC materials contain postnatal data specimens at ~0, 3, and 12 months, in addition to adult and elderly. MBRC does not yet have materials at 48 months.

## 5 CONCLUSIONS

Our conclusions are simple: (1) we underline the absolute importance of the rhesus macaque as an animal model for neuroscience research, while recognizing the importance of other animal models. (2) We advocate increased use of archived materials and their sharing. We are convinced that this will decrease animal sacrifice and research costs while increasing study transparency, repeatability, and efficiency. (3) We promote the use of both M and F specimens to be able to evaluate sex as a biological variable, and (4) we encourage the use of different ages to understand normal and abnormal changes during the lifespan, from early fetal development to elderly. We trust that the richness of the data available in the MBRC Collections complements other public datasets and that, together, they more accurately reflect normal human brain differences due to sex and age and permit better comparisons to lesion, treated, and pathological conditions. In turn, we expect that these efforts increase scientific rigor and expedite further advances in neuroscience research more conducive to translational medicine with true practical applications in the clinical setting.

## AUTHOR CONTRIBUTIONS

All authors contributed to editorial changes in the manuscript. All authors read and approved the final manuscript. VMS, LG, EB, CC, JC, KK, AN, and BA are currently using MBRC materials to conduct research with or under the direction of AD, PR, ZM, JLL, NK, KSR, and JIA. AD, VMS, EB, CC, KK, and LG searched and summarized available literature and wrote the paper. PB develops, manages, maintains, and supports MBRC technology under the direction of AD. JLL, KR, NK, DD, JIA, AFTA, YMM, CJZ, BA, and ZM have active research collaborations with AD and use MBRC materials on a regular basis. LG, EB, and CC scanned materials and provided microscopy. YMM performed EM. AD and PR provide funding. AD is the director of the MBRC.

## FUNDING INFORMATION

The MBRC is supported by NIH grant MH113257 (AD). Many of the materials that became part of the Collections in the MBRC were obtained in part under DA023999 (PR). Procurement and processing of some of the materials in Collection 8 was funded through the Yale Alzheimer Disease Research Center (1P30AG066508, PI: Strittmatter, S). Dibyadeep Datta was funded by 1R21AG079145‐01, KL2 TR001862, Alzheimer's Association Research Grant AARGD‐23‐1150568, Alzheimer's Association and National Alzheimer's Coordinating Center (NACC) New Investigator Award NIAP25‐1445690 and P30AG066508 Developmental Project Award.

## CONFLICT OF INTEREST STATEMENT

The authors declare no conflict of interest.

## ETHICS STATEMENT

No animals were sacrificed solely for the present study. All animals whose brains became part of the MBRC brain bank, and which were processed to obtain the datasets hereby used, were for unrelated studies. All those studies were previously reviewed and approved by the Institutional Animal Care and Use Committee (IACUC) of Yale University. Animal welfare is as specified in the Guide for the Care and Use of Laboratory Animals and in accordance with Veterinary Medical Association Guidelines. Human tissue is only present in Collection 8. The samples are non‐identifiable biological specimens exempt from Institutional Review Board approval. Human tissue was assessed from decedents enrolled through the Yale ADRC.

## DECLARATION OF AI AND AI‐ASSISTED TECHNOLOGIES IN THE WRITING PROCESS

AI tools were not used in drafting of the manuscript. AI tools, specifically Aiforia and QuPath, are being used for image analysis in MBRC projects. Machine learning and AI tools are being explored because they offer a unique opportunity to collect, process, and analyze large datasets in an unbiased and efficient manner.

## Data Availability

See: https://medicine.yale.edu/neuroscience/macbrain/. Available specimens in Collections 1–5 can be viewed on the website and physically accessed by request. Glass slides in collections 1–4 continue to be refurbished as needed by removing coverslips and adding new mounting media and cover glasses. This is to replace the oftentimes polymerized old mounting media. EM blocks from Collection 5 are available to interested researchers, and the MBRC is able to assist in processing and providing ultrathin sections instead of complete blocks. This not only expedites studies but also assures increased usefulness and resource availability. In Collections 6 and 8, each series of sections appears as a gallery that can be viewed on any device with access to the internet, including smart phones and tablets. All images are zoomable and downloadable (see: https://macbraingallery.yale.edu/collection6/). In addition, when presenting data to the public, we provide a QR that gives immediate access to the MBRC data sets. Upon request, frozen tissue blocks in Collection 7 can be sent to researchers depending on availability, as per research requirements (e.g., a particular brain region of a M or F animal of a specific age). Besides digital access via the internet, data can also be viewed on site by arranging a visit to our location in the Department of Neuroscience at Yale University School of Medicine.
